# Effects of cooperative learning on students’ learning outcomes in physical education: a meta-analysis

**DOI:** 10.3389/fpsyg.2025.1508808

**Published:** 2025-05-13

**Authors:** Hulusi Boke, Yalin Aygun, Sakir Tufekci, Fatma Hilal Yagin, Burak Canpolat, Goktug Norman, Pablo Prieto-González, Luca Paolo Ardigò

**Affiliations:** ^1^Yasar Oncan Secondary School, Ministry of National Education, Malatya, Türkiye; ^2^Department of Sport Management, Faculty of Sport Sciences, Inonu University, Malatya, Türkiye; ^3^Department of Biostatistics, Faculty of Medicine, Malatya Turgut Ozal University, Malatya, Türkiye; ^4^Department of Physical Education and Sport on Disabilities, Faculty of Sport Sciences, Inonu University, Malatya, Türkiye; ^5^GSD-HPE Department, Sport Sciences and Diagnostics Research Group, Prince Sultan University, Riyadh, Saudi Arabia; ^6^Department of Teacher Education, NLA University College, Oslo, Norway

**Keywords:** cooperative learning, classroom physical activities, learning outcomes, meta-analysis, physical education

## Abstract

This meta-analysis examines the effect of Cooperative Learning (CL) interventions, compared to traditional instructional methods, on students’ learning outcomes across affective, cognitive, physical, and social domains in physical education (PE). The review involved a comprehensive search of 12 databases in English, Spanish, and Turkish, with the last search conducted on June 2nd, 2024. Studies included were true experimental or quasi-experimental designs featuring direct CL interventions in PE, covering students of both genders from primary school to university levels. The standardized Cochrane methods were used to identify eligible records, collect and combine data, and assess the risk of bias. Comprehensive Meta-Analysis (CMA) v4 software package was used to yield a summary of quantitative results. Hedges’s *g* was used as the effect size (ES) measure, calculated from pre- and post-tests in both experimental and control groups. Forty-three studies (comprising 60 reports) were initially included, but three studies were excluded as outliers, leaving 40 studies (56 reports) with a total of 3.985 participants for analysis. The random effects model revealed a moderate positive overall effect of CL interventions (ES = 0.459, 95% CI = [0.324, 0.592], *p* < 0.001), indicating that CL enhances PE students’ learning across four domains. Subgroup analyses showed small to moderate ESs for affective (ES = 0.304), physical (ES = 0.471), cognitive (ES = 0.589), and social learning (ES = 0.612). Risk of bias was evaluated using Begg and Mazumdar’s rank correlation, the classic fail-safe number, and a funnel plot, all indicating a low risk of bias. Methodological quality was assessed using the Medical Education Research Study Quality Instrument (MERSQI). The study was registered on PROSPERO (ID: CRD42024532607). This meta-analysis underscores the effectiveness of CL as a student-centered pedagogical model in PE, demonstrating its positive effect on various learning outcomes in the affective, cognitive, physical, and social domains. The findings provide instructive data and strategies for researchers, practitioners, and policymakers aiming to integrate, implement, or make context-specific adaptations of CL into educational processes, while ESs in the affective, physical, cognitive, and social learning domains provide domain-based implementation guidance for these stakeholders.

## 1 Introduction

Cooperative Learning (CL) emerged in the mid-1970s at Johns Hopkins University (Slavin, 1990) as an innovative pedagogical practice aimed at helping students at various grade levels to cultivate shared intellectual development (Johnson and Johnson, 1999). The central tenet of CL is shared success rather than just sharing success (Slavin, 1995, 1996; Johnson and Johnson, 1999, 2009; Zhou et al., 2023) as it reduces social hierarchies and individual conflicts, focuses on collective responsibility and minimizes competition within and between students.

Although CL, as a pedagogical model, has a deep-rooted epistemological heritage in the educational sciences, in recent years it has become an interdisciplinary research focus in the physical education (PE) landscape (Fernandez-Rio and Iglesias, 2024), with the rise of student-centered model-based practices (Casey and Kirk, 2024) and the academic prioritization of the holistic development paradigm (Bailey, 2009; Casey and Kirk, 2024). Last two systematic reviews by Casey and Goodyear (2015) and Bores-García et al. (2021) have shown that the CL model has an important place in PE classes, promoting a range of positive learning outcomes for students. We propose that the CL model in PE, compared to traditional instructional methods, has a statistically significant effect on students’ learning outcomes in the affective, cognitive, physical, and social domains. Furthermore, social, demographic, and assessment factors may moderate the link between CL and learning outcomes. We test this theoretical claim via a meta-analysis of 40 studies (56 effect sizes [ES]).

## 2 Literature review

### 2.1 The application of CL in PE

As teaching and learning PE in schools in the 21st century still poses a significant challenge (MacPhail and Lawson, 2020), epistemological and pedagogical debates question the best way of organizing the interdependent elements of curriculum, learning and teaching practices to achieve specific learning outcomes (O’Neil and Boyce, 2018; Murtagh et al., 2023; Fernández-Vázquez et al., 2024; Howley et al., 2024). This inquiry began with a resistance to the didactic hegemony of Mosston (1966) traditional Teaching Styles; since the 1970s, structures have evolved from traditional teacher-centered approaches to novel student-centered models (Casey, 2016; Casey and Kirk, 2024). This educational evolution was triggered by Joyce and Weil’s (1972) Instruction Models, which centered on the active participation of the learner, creating an epistemic break; and by the Curriculum Models formulated by Jewett and Bain (1985), which codified interdisciplinary view as an ontic necessity, moving the paradigm to a pluralist axis. Metzler (2000) consolidated this process theoretically with the Instructional Models that operationalized the episteme of social constructivism, while Haerens et al. (2011) and Kirk (2013) evolved the paradigm from linear pedagogy to a rhizomatic matrix with Pedagogical Models. As a manifesto of the evolution of Models-Based Practices (Casey and Kirk, 2024), Kirk (2013) call for educationally beneficial learning outcomes for students in four domains (affective, cognitive, physical, social), gained empirical reinforcement in an umbrella review conducted by Fernandez-Rio and Iglesias (2024). The study reveals that the most widely recognized, practiced, and investigated pedagogical models (Casey and Kirk, 2024), such as Sport Education (SE), Teaching Games for Understanding (TGfU), Teaching Personal and Social Responsibility (TPSR), and CL (the model under investigation) despite different features of each, support the comprehensive and coherent learning achievements, with strong evidence embodying Kirk’s theoretical argument. Table 1 systematically presents a synthesis of the main advantages, limitations and ESs of these pedagogical models.

**Table 1:** Summary of the strengths, weaknesses, and reported ESs of common pedagogical models in PE.

Pedagogical models	Strengths	Weaknesses	ESs
SE	Multi-dimensional learning (e.g., game performance, teamwork, self-efficacy, fair play); structures similar to real sports environment; increasing student motivation and engagement (Siedentop, 2001; Siedentop et al., 2019).	The need for long-term planning and teacher training; formal competition favors high ability students; content diversity and the challenge of continuous assessment (Araújo et al., 2014; Fernandez-Rio and Iglesias, 2024).	Autonomy (ES = 0.43), competence (ES = 0.42), relatedness (ES = 0.57), intrinsic motivation (ES = 0.63), prosocial attitudes (ES = 0.46; Manninen and Campbell, 2022).
TGfU	Developing tactical understanding, decision-making and game strategies; acquiring technical skills in a meaningful context; supporting problem solving and cognitive development (Bunker and Thorpe, 1982; Miller, 2015; Harvey et al., 2018).	Dependence on time, infrastructure and poor performance of student-coaches; inability to complete tactics-oriented training in a short period of time; complexity of implementation of the game-based approach (Harvey and Jarrett, 2014; Fernandez-Rio and Iglesias, 2024).	Decision making skills (ES = 5.93; Ortiz et al., 2023).
TPSR	Integrating personal and social responsibility skills with physical activity practices; life skills for low-income students; transferable values education (Hellison, 2011).	Sustainability of values education; lack of systematic assessment for behavior change; insufficient time for social responsibility in school curricula (Pozo et al., 2018; Shen et al., 2022; Fernandez-Rio and Iglesias, 2024).	Social-affective learning (ES = 0.37; Aygun et al., 2024); personal responsibility (ES = 0.38), social responsibility (ES = 0.20; Sánchez-Miguel et al., 2025).
CL	Social-affective learning and empathy development; strengthening group work and decision-making; promoting self-management and responsibility (Slavin, 1990; Johnson and Johnson, 1999; Casey and Goodyear, 2015; Dyson and Casey, 2016; Bores-García et al., 2021).	Difficulty in structuring collaborative tasks and managing group dynamics; need for teacher guidance and monitoring individual responsibility; time and planning intensive (Casey and Goodyear, 2015; Bores-García et al., 2021; Fernandez-Rio and Iglesias, 2024).	Intrinsic motivation (ES = 0.38; Liu and Lipowski, 2021); self-determined motivation (ES = 0.95; Sierra-Díaz et al., 2019)

SE, Sport Education; TGfU, Teaching Games for Understanding; TPSR, Teaching Personal and Social Responsivity; CL, Cooperative Learning.

The core concept underlying SE is to develop competent, literate and enthusiastic boys and girls through authentic sport experiences strategically designed around six structural features (Siedentop, 2001): seasons, affiliation, formal competition, culminating event, record keeping and festivity (Siedentop et al., 2019). The crux of TGfU is to radically reverse the ‘technical skills first’ paradigm by utilizing modified game play (representation and exaggeration; Mitchell et al., 2013) to create a cognitively demanding learning environment in which students can explore the deeper tactical dynamics of the game play (e.g., zone reading, decision-making chains, collective action strategies; Bunker and Thorpe, 1982; Harvey et al., 2018). The central tenet of TPSR is to provide underserved children with the opportunity to develop personal and social responsibility skills through physical activity practices (Hellison et al., 2000) to apply these skills in other social settings, and to develop a sense of responsible citizenship (Hellison, 2011). CL was designed to unleash the dialectical synergy of learning in collective dialog (with each other), autonomous-interactional agency (by each other) and around common ontological goals (for each other; Dyson and Casey, 2016). This point of view transforms educational practice into a radical interaction by combining the symbiotic relationship of stimulating interactions along the axis of positive interdependence, the dialectics of interpersonal skill dynamics with collective autonomy, the articulation of process-oriented cognitive processing mechanisms with ontological responsibility, and finally the constitutive role of individual accountability within this organic totality (Johnson et al., 1994), all within a context of methodological-epistemological coherence. In this context, CL transforms educational practice into an ontological whole by weaving affective, cognitive, physical, and social layers together (Casey and Goodyear, 2015; Bores-García et al., 2021) and has a great research interest (74 studies superior to TGfU [49 studies] and TPSR [47 studies]; Iglesias et al., 2023).

The existing meta-analyses findings of CL (intrinsic motivation: ES = 0.38; self-determined motivation: ES = 0.95) address its pedagogical effectiveness with an ontological narrowing limited to the affective domain (Sierra-Díaz et al., 2019; Liu and Lipowski, 2021). Connected to this gap, this study develops the first meta-analytic model that reinterprets the mechanisms of CL’s hegemonic influence through the interdisciplinary dialectics of different learning outcomes in four domains (affective, cognitive, physical, social) into a triad of interdisciplinary synergy, epistemological continuity and ontological integrity.

In ‘liquid times’ writings, Bauman (2007), while analyzing contemporary society shaped by spatial and temporal dismemberment, touches on similar themes as other authors (e.g., Giddens, 1991, 1998; Beck, 1992, 1998; Nowotny, 1994; Sennett, 1998; Harvey, 2005), he positions the global age in a darker perspective with the concept of ‘liquid modernity’. Bauman portrays the constant liquefaction of social norms and relations as a dark ontological break where individuals are forced to adapt to this unstable reality. In this context, CL in PE can offer a resilient pedagogical alternative to the fragmentation created in today’s world by the ‘liquid modernity’ that Bauman criticizes. CL’s social structure based on the principle of students learning with, for, and through each other; its collective goal-oriented activities and peer-assessed processes (Dyson and Casey, 2016) can build a socio-pedagogical field of resistance against the individualism of the ‘liquid modernity’. CL, the most studied model in the PE literature after SE (Fernandez-Rio and Iglesias, 2024), also reveals an important shortcoming: the lack of a comprehensive meta-analysis that critically evaluates its product-oriented outcomes. This shortcoming constitutes the main motivation of our study.

CL is a synergistic pedagogical model in PE underpinned by multiple theoretical frameworks (Dyson and Casey, 2016; Metzler, 2017). Johnson and Johnson’s (2009) Social dependency theory emphasizes students’ collective efforts toward common goals (positive social dependency), while Vygotsky (1978) zone of proximal development theory explains the mentoring role of skilled peers in motor skill transfer. Bandura’s (1986) social cognitive theory explains how students gain self-confidence and motivation through peer observation. The constructivist approach (Bruner, 1997) supports the social construction of knowledge through group strategy discussions. Self-determination theory (Deci and Ryan, 2000) explains how CL fosters intrinsic motivation through autonomous role allocation and peer feedback. Motor learning theories (Schmidt, 1975; Newell, 1985) legitimize the collective analysis of teamwork challenges.

The main idea behind CL in PE is to teach students to achieve dynamic, creative, co-experiential learning outcomes through high-quality physical activity practices and sports (Dyson and Casey, 2016; Metzler, 2017). Thus, students experiencing CL applications in PE usually exhibit positive perceptions of the classroom atmosphere (Fernandez-Rio et al., 2017), which accentuate their subjectivity and personal inclusivity. Furthermore, as an effective counterweight to Liquid Modernity’s attribution of isolation and uncertainty, CL equips students with a range of authentic, meaningful, and transferable life skills (Lee, 2014; Dyson and Casey, 2016; Metzler, 2017). Physical fitness (Lee, 2014), social responsibility (Casey and Goodyear, 2015; Bores-García et al., 2021), critical thinking (Huang et al., 2017), motivation and mutual encouragement (Fernández-Espínola et al., 2020; Montoya et al., 2020; Liu and Lipowski, 2021), fairness and inclusivity (Cañabate et al., 2021), and communication and teamwork commitment (Sánchez-Hernández et al., 2018) have been well documented. Particularly, the CL application in PE can empower students to transcend the passive role of observers, a common pitfall in PE classes (Zhou et al., 2023). This principle holds true for the pedagogical landscape of PE across diverse educational tiers, spanning from primary/elementary school (Dyson, 2001) to secondary/middle/high school levels (Casey, 2013), and extending to university settings (Cohen and Zach, 2013).

At present, by leveraging the possibilities provided by digital technologies (Jastrow et al., 2022; Wang et al., 2023), ongoing scholarly investigations, practical applications, and collaborative networks, the CL model can be strategically integrated in synergy with other pedagogical models in PE (e.g., hybridization of the SE model; Fernandez-Rio and Casey, 2021) and offer customizable flexibility to different PE contexts. This integration demonstrates the potential of CL to create a multi-layered and synergetic learning atmosphere that foster students’ all-around development while expanding its worldwide recognition and acceptance (Dyson and Casey, 2016; Zhou et al., 2023).

The CL’s effectiveness in PE is embodied in structured and interactive teaching strategies such as Student Teams-Achievement Divisions (STAD), Learning Team (LT), Teams-Games-Tournament, Think-Pair-Perform, and Jigsaw (Metzler, 2017; Dyson et al., 2022). These strategies allow teachers to adapt learning objectives to the specific requirements of the curriculum and to design pedagogical applications that are sensitive to the individual needs of students. Furthermore, as the foundation of CL, interpersonal skills, processing, positive interdependence, promotive interaction, and individual accountability (Johnson et al., 1994) serve as scaffolding that help students to achieve specific learning outcomes in PE (Goodyear et al., 2014; Casey and Goodyear, 2015; Bores-García et al., 2021; Zach et al., 2023). The successful application of CL not only empowers learners to exercise autonomous agency in the cultivation of self-directed learning trajectories but also strategically positions them as active co-architects in the orchestration of collaborative achievement, thereby engendering a dialectical interplay between individual metacognitive development and collective knowledge synthesis.

### 2.2 Previous reviews

The existing literature has addressed the role of CL in the context of PE through unidimensional meta-analyses, systematic reviews, bibliometric analyses, and regional studies. While learning outcomes in four domains (affective, cognitive, physical, social) are brought up as legitimate product outcomes in PE (Bailey, 2009; Casey and Kirk, 2024), a systematic review by Casey and Goodyear (2015) revealed a scarcity of quantitative synthesis in a synoptic examination of the effects of CL on learning outcomes in these four domains. Although the study empirically confirmed that CL encourages authentic and transformative learning outcomes in the four domain, the fact that the learning outcome data in the affective domain remained only anecdotal and the learning in these four domains were not explored by quantitative syntheses such as ES prevented a complete understanding of the pedagogical potential of the model. Bridging this research gap could make critical contributions to the development of evidence-based curriculum designs and shaping global physical activity policies, as well as reinforcing the theoretical validity of CL. Six years later, Bores-García et al. (2021) expanded the scope of the literature by analyzing studies between 2014 and 2019 with geographical context, demographic trends and authorship dynamics. However, by adopting a descriptive approach and ignoring measures of ES (also as in Casey and Goodyear’s review), they hampered the understanding of the tangible effects of CL. This weakens the evidence base to support pedagogical decisions.

Regarding meta-analysis development, only three comprehensive studies represent a typical reflection of the reductionist and fragmented point of view in the literature, focusing on the effects of CL on intrinsic motivation (Fernández-Espínola et al., 2020; Liu and Lipowski, 2021) and self-determined motivation (Sierra-Díaz et al., 2019) on the axis of affective learning outcomes. While these studies present results on the effectiveness of CL interventions that exhibit methodological consistency but weaknesses in terms of comprehensive representativeness, the exclusion of an integrative perspective on multidimensional learning outcomes such as cognitive, physical, social domains and components of the affective domain (due to the singularity-focused research paradigm) reveals a critical gap in the conceptualization of CL as a pedagogical model. In this regard, our study attempts to strengthen CL’s model claim through quantitative synthesis by combining learning outcomes in a balanced way across affective, cognitive, physical, and social domains. Thus, we clarify how CL can contribute to holistic educational goals beyond the existing evidence limited to ‘single domain’ meta-analyses.

Dyson et al. (2022) provided a contextual perspective by examining cultural adaptations of CL in Chinese PE curricula. However, their avoidance of cross-cultural comparisons and lack of quantitative synthesis limited the generalizability of their findings. Zhou et al. (2023) mapped the trends in the research themes of CL through bibliometric analysis of 169 studies, but could not make a meta-analytic contribution due to their focus on pattern identification rather than statistical integration. While these studies contributed to the understanding of CL on a global scale, they were insufficient in providing practical guidance as they did not fill quantitative gaps. Most recently, Zach et al. (2023) examined the relationships between CL constructs and students’ learning outcomes in PE using discriminant analysis and illuminated operational dynamics. However, their avoidance of meta-analysis suggests that they missed the opportunity to create cumulative ES metrics. Even such innovative methodologies should be supported by meta-analytic rigor to measure causal relationships or pedagogical effectiveness.

In the existing literature, reviews examining the pedagogical effects of the CL model in PE have not been able to fully reveal the multidimensional potential of the model due to significant methodological and conceptual limitations. Among these limitations, the lack of quantitative synthesis of the learning outcomes in the affective, cognitive, physical and social domains and comparative analysis of these domains’ ES stand out. Moreover, the lack of meta-analytic frameworks limited the integration of cumulative results and statistical model development of interactions across domains. Moreover, the lack of additional analyses of how moderator variables (country, grade level, intervention duration, etc.) shape the effects of CL has made it difficult to understand the context-sensitive dynamics of the model. All these shortcomings may significantly hamper a comprehensive mapping of the pedagogical mechanisms of CL in PE and the development of high-quality evidence-based practices.

### 2.3 Value of meta-analysis

Meta-analysis is a statistical method that synthesizes results by aggregating study ESs and their variances, providing a quantitative summary of results (McKenzie and Brennan, 2019). This meta-analysis study aimed to show a quantitative summary of results on the effect of the CL model on students’ learning outcomes in the affective, cognitive, physical and social domains within PE context. The study not only comparatively reveals in which developmental domains CL is more effective (e.g., the relative superiority of social ES over affective ES), but also aims to explain the underlying dynamics of pedagogical mechanisms by analyzing ESs from the view of causal relationships. Thus, three broad research questions (RQs) were developed. RQ1: What is the effect of CL on students’ learning outcomes in the affective, cognitive, physical, and social domains in PE? RQ2: When examining the hierarchical effects of CL on students’ learning outcomes in a cross-domain analytical framework (affective, cognitive, physical, social), how does the measurable effect trend? RQ3: How are the ES of CL affected by moderator variables such as publication source, country, grade level, research design, sport content, teaching structure, intervention duration (weeks), publication year, sample size and study quality score (a unique variable)?

## 3 Materials and methods

### 3.1 Registration and protocol

This meta-analysis was registered in International Prospective Register of Systematic Reviews (PROSPERO), an international database for systematic reviews in health and related fields, under the registration number CRD42024532607 (Schiavo, 2019). The protocol was developed based on the Preferred Reporting Items for Systematic Reviews and Meta-Analyses (PRISMA) statement (Page et al., 2021) for complete, accurate, and transparent reporting.

### 3.2 Eligibility criteria

The PICOS (Population, Intervention, Comparison, Outcomes, and Study) framework was utilized to develop precise inclusion and exclusion criteria (see Table 2; Amir-Behghadami and Janati, 2020). In order to minimize selection bias in line with Cochrane guidelines (Higgins et al., 2024), studies published in 2000 and later were included. This date represents the period when CL gained methodological consistency in PE and proved its applicability on a global scale (Casey and Goodyear, 2015). Although year of publication was added as a moderator variable to capture model-based developmental changes, temporal heterogeneity did not show statistical significance in preliminary analyses (see, Table 3). Thus, the 25-year timeframe covers the evolution of the pedagogical model while maintaining methodological robustness.

**Table 2:** The PICOS framework for eligibility criteria.

PICOS tool	Inclusion criteria	Exclusion criteria
P	Students of both genders across all educational levels, including primary, secondary, high school, and university.	Non-students (e.g., teachers, educators, youth workers, coaches, athletes, etc.). Students who are not related to PE.
I	Direct CL intervention in PE regarding the achievement of learning across the affective, cognitive, physical, and social domains. Both short and long CL interventions.	Hybridization of CL with other pedagogical models and methods for intervention.
C	Traditional (teacher-centered) and competitive pedagogical models and methods, PE curriculums.	Pedagogical models and methods addressing cooperative or collaborative approaches (e.g., peer-assisted learning).
O	Statistical data necessary for its calculation, including mean, standard deviation, sample size for both the experimental and control groups.	Lack of crucial statistical data, such as the mean, standard deviation, sample size for both the experimental and control groups, poses significant challenges.
S	ED or QED, each with two groups.	Single-group cross-sectional or single-group pretest and post-test or post-test only.

CL, cooperative learning; PE, physical education; ED, true experimental design; QED, quasi-experimental design.

**Table 3:** CL and learning outcome: univariate analyses of variance for moderator variables (categorical).

	Parameter	Estimate	SE	Z-value	95% CI for B
Duration of intv. (in weeks)	β_0_	0.508	0.146	3.48	[0.222, 0.795]
	β_1_	−0.007	0.012	−0.68	[−0.032, 0.017]
	Q_Model_ (1, k = 52) = *p* > 0.05				
Year of publication	β_0_	15.31	24.690	0.62	[−33.074, 63.709]
	β_1_	−0.007	0.012	−0.60	[−0.031, 0.016]
	Q_Model_ (1, k = 54) = *p* > 0.05				
Sample size	β_0_	0.493	0.113	4.34	[0.270, 0.716]
	β_1_	−0.001	0.001	−0.69	[−0.002, 0.001]
	Q_Model_ (1, k = 52) = *p* > 0.05				
Q score	β_0_	−0.355	0.985	−0.36	[−2.286, 1.576]
	β_1_	0.057	0.072	0.80	[−0.083, 0.199]
	Q_Model_ (1, k = 52) = *p* > 0.05				

SE, standard error; CI, confidence interval.

### 3.3 Search strategy

The search strategy used descriptors such as the primary term ‘cooperative learning’ in conjunction with subcategories including ‘school,’ ‘class,’ ‘physical education,’ ‘physical activity,’ and ‘movement.’ The inclusion of ‘physical activity’ and ‘movement’ as related terms aimed to refine the scope of studies to those that, while reporting on CL in PE, also draw on contexts involving physical activity practices and movement to enrich the PE literature. Following this initial search, the identified studies were scrutinized for their relevance and suitability.

### 3.4 Information sources

The meta-analysis incorporated studies from diverse sources to ensure comprehensive analysis. The included literature comprised published articles, master theses, and doctoral dissertations in English, Spanish, and Turkish languages. The studies in English were sourced from ERIC, Google Scholar, ProQuest Dissertations and Theses, Psych ARTICLES, Psych INFO, Sport DISCUS, and WOS databases. The studies in Spanish were sourced from Dialnet, RECYT (Spanish Repository of Science and Technology), and TESEO (Consultation of the Doctoral Theses) databases. Sources in Spanish, in addition to English and Turkish, were included because, according to the review by Zhou et al. (2023), Spain has produced the highest number of publications on CL in PE, with 71 papers cited up to 404 times (Zhou et al., 2023). Spanish was the primary language of one of the authors (P.P.-G.) in this review. The studies in Turkish were sourced from ULAKBIM (Turkish Academic Network and Information Center) and YOK (Turkish Higher Education Council) Doctoral and Master Theses databases. Turksih was the primary language of six of the authors (H.B., Y.A., S.T., F.H.Y., B.C., and G.N.). Each source was last searched or consulted on June 2nd, 2024.

Although conference proceedings are widespread in the academic literature, the data shared in these sources exhibit significant heterogeneity in terms of methodological reliability, data accuracy and level of detail, making their inclusion in meta-analysis methodologically risky (Liu et al., 2017). Therefore, conference abstracts were excluded to maintain the internal validity of the analysis.

### 3.5 Selection process

Three authors (H.B., Y.A., and P.P.-G.) independently reviewed the titles and abstracts of the initial records and resolved any discrepancies through discussion until they reached a consensus. Subsequently, the authors worked in pairs to independently screen the titles and abstracts of all individual studies. In instances of disagreement, consensus regarding which studies to assess in full-text was reached through deliberation. This comprehensive review process aimed to verify the eligibility of each study according to the criteria for inclusion and exclusion detailed in Table 2. If needed, Y.A. was consulted for the ultimate decision-making authority. Subsequently, two authors (H.B. and P.P.-G.) conducted independent assessments of the full-text studies for inclusion (Waffenschmidt et al., 2019; Wang et al., 2020). Again, in cases of disagreement, consensus on inclusion or exclusion was reached through deliberation, with the Y.A. being consulted if needed (Bomhof-Roordink et al., 2019). The inter-coder reliability index for Cohen’s Kappa was computed as 0.86 (Cohen, 1960; Landis and Koch, 1977; Viera and Garrett, 2005), demonstrating substantial agreement.

### 3.6 Data collection process

A study-specific data extraction template was utilized for the data collection process. Two review authors (H.B. and P.P.-G.) employed this template to methodically extract data from eligible studies. The extracted data were systematically compared, with any discrepancies resolved through deliberation. Ş.T. then entered the data into EXCEL spreadsheet with various data items, ensuring accuracy through double-checking.

### 3.7 Data items

Data were collected regarding the author, country, grade level, intervention characteristics, learning outcomes in the affective, cognitive, phsyical, and social domains, participant characteristics, research design and features, source of publication, sports content, teaching structure, year of publication, and study quality score. Eligible outcomes were broadly categorized as learning outcomes in the four domains (affective, cognitive, physical, social). Any quantitative assessment of learning outcomes was eligible for inclusion.

The identification and standardization of data to be used in meta-analyses is a critical process. As emphasized by Cochrane (Higgins et al., 2024), these data are often reported in heterogeneous formats (mean, standard deviation, ES) and require methodological harmonization prior to analysis. In this study, in order to quantitatively synthesize the effect of the CL model on various learning outcomes in the affective, cognitive, physical and social domains, data on the sub-dimensions of each domain (e.g., motivation, self-efficacy, critical thinking, problem solving, psychomotor abilities, empathy, peer relations) were integrated in a systematic approach through a data combination formula proposed by Cochrane (Higgins et al., 2024). Inverse-variance weighting method, for continuous or dichotomous outcomes was used. The standardized z-scores calculated for each of these sub-dimensions were adopted as the basis for determining the combined ES of the respective domains. The contribution of each sub-dimension was balanced with weighted averages to derive an overall domain-specific effect value. This process ensured methodological consistency and increased comparability across studies.

### 3.8 Risk of bias assessment

**Figure 1:**
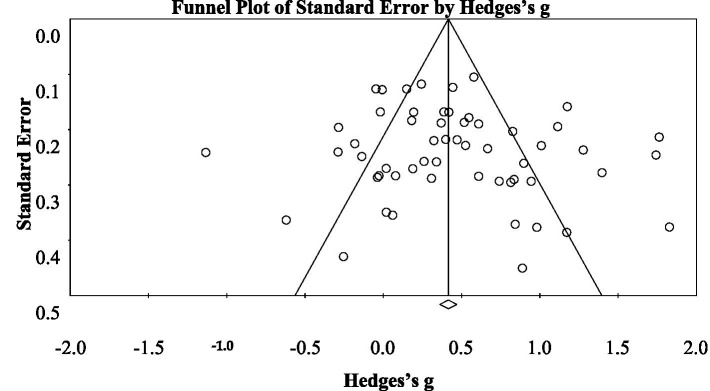
Funnel plot of ESs of the betas between CL and learning outcome.

Risk of bias in the result of the synthesis due to missing studies or results within studies was considered (Page et al., 2019). To assess small-study effects and bias in the included studies, a funnel plot was generated (refer to Figure 1). Additionally, the classic fail-safe number (Nfs) method (Khoury et al., 2013), Begg and Mazumdar (1994) rank correlation, and Egger et al.’s (1997) regression intercept were utilized. Two independent reviewers, H.B. and Y.A., conducted assessments for each study to identify potential errors in evaluations (Robson et al., 2019). They independently appraised the risk of bias and provided justifications for their judgments. Any inconsistencies in their assessments were resolved through deliborations, ensuring a consensus was reached.

#### 3.8.1 Study quality assessment

The methodological rigor and reporting quality of each included study were appraised using the Medical Education Research Study Quality Instrument (MERSQI; Reed et al., 2007). The MERSQI involves 10 specific criteria organized into six primary dimensions: study design, sampling methods, data collection procedures, validity of assessment instruments, data analysis techniques, and outcomes assessment. In alignment with the PE context, the MERSQI was refined to specifically omit the assessment of potential healthcare-related outcomes. Following revisions, the instrument achieved a maximum score of 17, contrasting with the original scoring range of 5 to 18. The comprehensive methodological quality scores for each study are delineated in Table 4. The average study methodological quality score for the included studies was assessed to be 13.56, with a range of 12 to 16.

**Table 4:** Characteristics of the 60 reports from 43 studies included in the meta-analysis.

Author (year)	Learning outcome^a^	ES	Source of publ.^b^	Country^c^	Grade level^d^	Spl. size	Res. desg.^e^	Teaching structure^f^	Sport content^g^	D. of intv. (in weeks)	Q
Abbood (2024)	3	−0.252	1	8	4	20	1	–	2	8	13.5
Aka (2014)	1	−0.180	2	11	5	84	2	4	11	10	13
Aka (2014)	2	−1.132	2	11	5	84	2	4	11	10	13.5
Aka (2014)	3	0.527	2	11	5	84	2	4	11	10	13.5
Altinkok (2012)	2	−0.016	2	11	3	139	2	1	9	12	13.5
Altinkok (2012)	3	0.196	2	11	3	139	2	1	9	12	13.5
Altinkok (2012)	3	−0.287	1	11	3	68	2	–	9	12	13
Bahadir (2011)	1	−0.135	2	11	4	63	2	1	11	8	12.5
Bahadir (2011)	4	1.399	2	11	4	63	2	1	11	8	13.5
Bayraktar (2011)	1	0.837	1	11	5	50	1	3	7	6	13.5
Bayraktar (2011)	2	0.947	1	11	5	50	1	3	7	6	14
Bayraktar (2011)	3	0.611	1	11	5	50	1	3	7	6	14
Bermejo Díaz et al. (2021)	4	0.310	1	10	5	55	–	–	9	14	14
Benkhaled et al. (2015)	3	0.080	1	1	2	48	1	–	9	8	13.5
Bensikaddour et al. (2015)	3	−0.026	1	1	4	48	–	3	9	12	12.5
Bofill-Herrero et al. (2022)	1	0.184	1	10	4	131	2	–	1	8	13
Cecchini Estrada et al. (2019)	1	0.581	1	10	4	372	2	1	4	24	14
Chiva-Bartoll et al. (2018)	1	0.401	1	10	4	96	2	–	8	8	13
Cochon Drouet et al. (2024)	3	−0.005	1	4	4	254	–	2	4	6	15.5
Darnis and Lafont (2015)	3	−0.618	1	4	3	30	1	–	4	10	13.5
Engels and Freund (2020)	1	0.421	1	5	1	142	2	5	9	14	15
Engels and Freund (2020)	4	0.389	1	5	1	142	2	5	9	15	16
Fadhul (2022)	1	0.845	1	8	5	30	1	–	6	4	13
Fadhul (2022)	3	0.062	1	8	5	30	1	–	6	5	13.5
Faozi et al. (2024)	3	0.669	1	7	2	75	1	4	2	1	14,5
Fernández and González-Mesa (2018)	1	0.021	1	10	3	31	2	4	–	6	12
Fernandez-Rio and Casey (2021)	4	1.744	1	10	2	90	2	–	4	6	14
Fernandez-Rio et al. (2017)	1	0.151	1	10	2	249	2	–	9	16	13
Fernandez-Rio et al. (2017)	4	−0.045	1	10	2	249	2	–	9.	17	14
Fernández-Río et al. (2014)	2	0.446	1	10	5	264	2	–	–	12	13.5
García-González et al. (2023)	4	0.246	1	10	4	286	2	–	9	5	15
Goudas and Magotsiou (2009)	4	0.611	1	6	4	44	–	1	4	6	13
Gulay (2008)	1	0.190	2	11	2	53	2	–	4	12	12.5
Gulay (2008)	4	0.020	2	11	2	53	2	–	4	12	13.5
Hu (2023)	1	0.743	1	2	5	48	2	–	10	24	12
Huang et al. (2017)	2	0.372	1	2	3	116	1	–	2	15	16
Huang et al. (2017)	3	0.519	1	2	3	116	1	–	2	15	16
Huang (2000)	3	1.116	1	2	5	120	1	–	9	10	14.5
Karavelioglu (2012)	3	0.325	2	11	5	82	2	2	6	22	13.5
Kiremitci and Dogan (2010)	2	1.830	1	11	4	39	–	–	5	10	12.5
Lafont et al. (2007)	3	0.891	1	4	3	20	1	–	2	10	13.5
Mohseen et al. (2011) *Out.*	4	7.454	1	8	4	60	–	–	11	12	13.5
Mostafa (2015)	3	0.983	1	3	4	30	1	2	2	30	13.5
Norito et al. (2019)	3	1.764	1	7	3	60	2	–	9	–	13
Palau-Pamies et al. (2022) *Out.*	1	9.344	1	10	4	185	2	4	1	2	13.5
Palau-Pamies et al. (2022)	2	1.177	1	10	4	185	2	4	1	2	14
Palau-Pamies et al. (2022) *Out.*	3	4.319	1	10	4	185	2	4	1	2	14
Pehlivan and Alkan (2010)	1	−0.284	1	11	4	103	–	3	3	7	13
Pehlivan and Alkan (2010)	3	0.829	1	11	4	103	–	3	3	7	13.5
Salih et al. (2021) *Out.*	3	6.279	1	8	5	60	1	–	8	9	14
Shalal et al. (2021)	3	1.174	1	8	5	30	1	–	2	6	13.5
Siong and Ali (2020)	3	0.899	1	9	4	63	2	1	1	4	13
Tuncel (2006)	2	1.279	2	11	4	85	2	1	2	11	13.5
Tuncel (2006)	3	0.473	2	11	4	85	2	1	2	11	13.5
Tuncel (2006)	4	1.012	2	11	4	85	2	1	2	11	14
Turan et al. (2023)	1	0.816	1	11	4	48	1	2	3	8	13
Uresin (2012)	1	−0.036	2	11	4	47	–	–	9	6	12
Winarni and Lutan (2020)	1	0.549	1	7	4	128	–	–	3	–	13
Yang et al. (2021)	1	0.341	1	2	4	59	2	–	2	6	12.5
Yang et al. (2021)	3	0.262	1	2	4	59	2	–	2	6	13

a 1 = affective, 2 = cognitive, 3 = physical, 4 = social.

b 1 = article, 2 = thesis.

c 1 = Algeria, 2 = China, 3 = Egypt, 4 = France, 5 = Germany, 6 = Greece, 7 = Indonesia, 8 = Iraq, 9 = Malaysia, 10 = Spain, 11 = Türkiye.

d 1 = combine, 2 = high school, 3 = primary school, 4 = secondary school, 5 = university.

e 1 = true experimental design, 2 = quasi-experimental design.

f 1 = combine, 2 = jigsaw, 3 = learning team (LT), 4 = student teams-achievement divisions (STAD), 5 = structural.

g 1 = badminton, 2 = basketball, 3 = curriculum, 4 = combine, 5 = dance, 6 = football, 7 = gymnastic, 8 = handball, 9 = physical condition, 10 = table tennis, 11 = volleyball.

### 3.9 Effect measures

Standardized mean difference ES and their corresponding 95% confidence intervals (CIs) were utilized for continuous data (Hedges and Olkin, 2014). Hedges’s g was selected as the ES measure, calculated using means, sample sizes, and standard deviations derived from both pre- and post-tests of the experimental and control groups. Initially, this meta-analysis included 60 reports from 43 studies: 13 true experiments, 21 quasi-experiments, and 9 studies with unknown classifications, all employing a two-group design. To quantify the magnitude of group differences in standat deviation units, Cohen’s d was employed for classification (Cohen, 1988).

### 3.10 Synthesis methods

Meta-analysis techniques and procedures, aimed at synthesizing study effect estimates and their variances to generate a quantitative summary of results (Deeks et al., 2019), were conducted using the Comprehensive Meta-Analysis (CMA) V4 software package (Borenstein et al., 2021) by two authors, H.B. and Y.A. Both the fixed-effect and random-effects meta-analysis models were also used in the study. The meta-analysis process was expanded to involve subgroup analyses examining learning outcomes in the affective, cognitive, physical, and social domains. Additionally, moderator analyses were conducted on six categorical variables: source of publication, country, grade level, research design, sport content, and teaching structure. Meta-regression analyses were also conducted on four continuous variables: duration of intervention in weeks, year of publication, sample size, and study quality score. These analyses aimed to elucidate the factors contributing to variability in results across included studies, with a specific focus on addressing statistical heterogeneity (Williams, 2012; Deeks et al., 2019). The presence of heterogeneity (variation in ES) across studies was assessed through inspection of a forest plot and calculation of Cochran’s Q statistic along with its corresponding *I^2^* statistic. Confidence in this metric was evaluated using 95% uncertainty intervals (CIs) around the *I^2^*. Thresholds of >75% were used to identify significant heterogeneity, with additional consideration given to the uncertainty intervals and the spatial distribution on the forest plot (Huedo-Medina et al., 2006; Page et al., 2018).

## 4 Results

### 4.1 Study selection

A total of 10.083 records were identified through database searching. After removing duplicates, 9.803 records remained for screening. 43 studies, including 36 peer-reviewed articles (Huang, 2000; Lafont et al., 2007; Goudas and Magotsiou, 2009; Kiremitci and Dogan, 2010; Pehlivan and Alkan, 2010; Bayraktar, 2011; Mohseen et al., 2011; Fernández-Río et al., 2014; Darnis and Lafont, 2015; Mostafa, 2015; Benkhaled et al., 2015; Bensikaddour et al., 2015; Altınkök, 2017; Fernandez-Rio et al., 2017; Huang et al., 2017; Fernández and González-Mesa, 2018; Chiva-Bartoll et al., 2018; Norito et al., 2019; Cecchini Estrada et al., 2019; Engels and Freund, 2020; Siong and Ali, 2020; Winarni and Lutan, 2020; Fernandez-Rio and Casey, 2021; Salih et al., 2021; Shalal et al., 2021; Yang et al., 2021; Bermejo Díaz et al., 2021; Fadhul, 2022; Palau-Pamies et al., 2022; Bofill-Herrero et al., 2022; García-González et al., 2023; Hu, 2023; Turan et al., 2023; Abbood, 2024; Faozi et al., 2024; Cochon Drouet et al., 2024), 2 master’s theses (Gulay, 2008; Uresin, 2012), and 5 doctoral dissertations (Tuncel, 2006; Bahadir, 2011; Altinkok, 2012; Karavelioglu, 2012; Aka, 2014), were reviewed and included in the meta-analysis, with each source cited accordingly. No additional studies meeting the inclusion criteria (see Table 2) were found. In total, 171 studies were excluded from the meta-analysis, comprising 8 theses and 125 peer-reviewed articles in English, 5 theses and 28 peer-reviewed articles in Spanish, and 3 theses and 2 peer-reviewed articles in Turkish. The rationale for their exclusion is elaborated upon Table 2. Initially (with outliers), this meta-analysis included 43 individual studies and their 60 reports, see Table 4.

**Figure 2:**
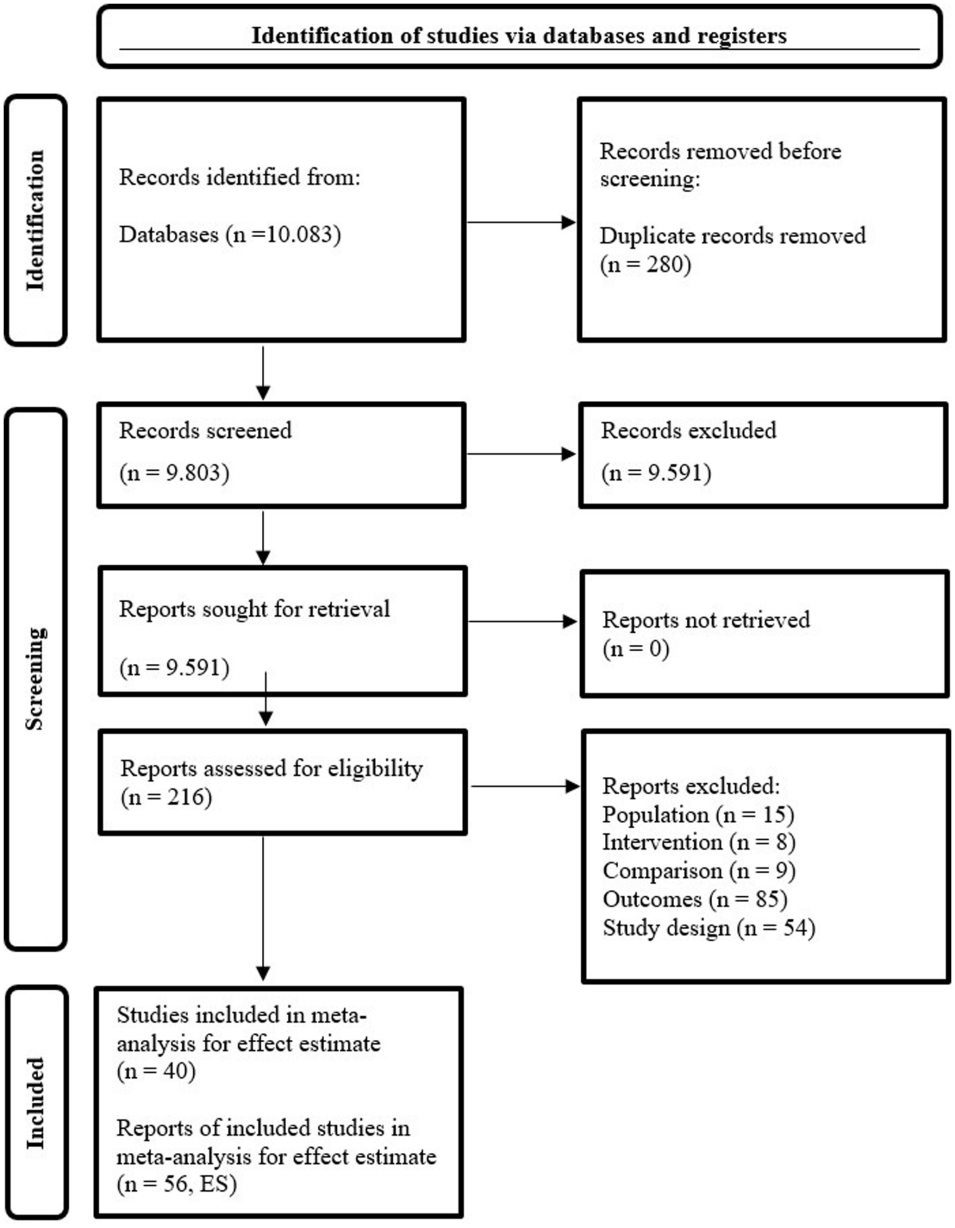
The PRISMA flow diagram for the meta-analysis (Page et al., 2021).

In order to prevent outliers from disproportionately affecting the analysis results in meta-analyses (Barnett and Lewis, 1994; Higgins et al., 2024), Hedges and Olkin (2014) stated that studies with standardized residuals outside the range ±3 can be considered as outliers. Accordingly, when some of the effects in the study were evaluated in terms of standardized residual values, it was determined that they deviated significantly from the pattern of the general dataset and their effects were quite high. The studies that were identified as outliers within the framework of the relevant criteria and excluded from the analysis are as follows: Mohseen et al. (2011), ES = 7.454; Palau-Pamies et al. (2022), ES = 9.344 and another effect from the same study, ES = 4.319; and Salih et al. (2021), ES = 6.279. Therefore, the ultimate meta-analysis stage comprised 56 reports from 40 studies, after excluding the outliers. The search and selection process is visually depicted through a flow diagram presented in Figure 2.

### 4.2 Study characteristics

Table 4 presents details for the included studies and their reports, including the learning outcome, ES, source of publication, country, grade level, sample size, research design, teaching structure, sport content, duration of intervention (in weeks), and study quality score.

### 4.3 Risk of bias

Upon excluding the outliers (see Table 4), funnel plot assessment and the collective analyses of Egger’s regression intercept, Begg and Mazumdar rank correlation, and Nfs consistently indicated a low likelihood of publication bias. Figure 1 illustrates a funnel plot demonstrating a predominantly symmetric distribution of the 56 effects, well within the expected range and centered around its axis. Egger’s regression intercept was 1.09, with a non-significant two-tailed *p*-value of 0.25. The rank correlation test reported a Kendall’s tau of 0.11 with a non-significant two-tailed p-value of 0.22. The Nfs value of 3,269 (*p* = 0.001) far exceeded the fail-safe criterion of 290 ([5 × 56] + 10; Figure 1).

### 4.4 Results of individual studies

**Figure 3:**
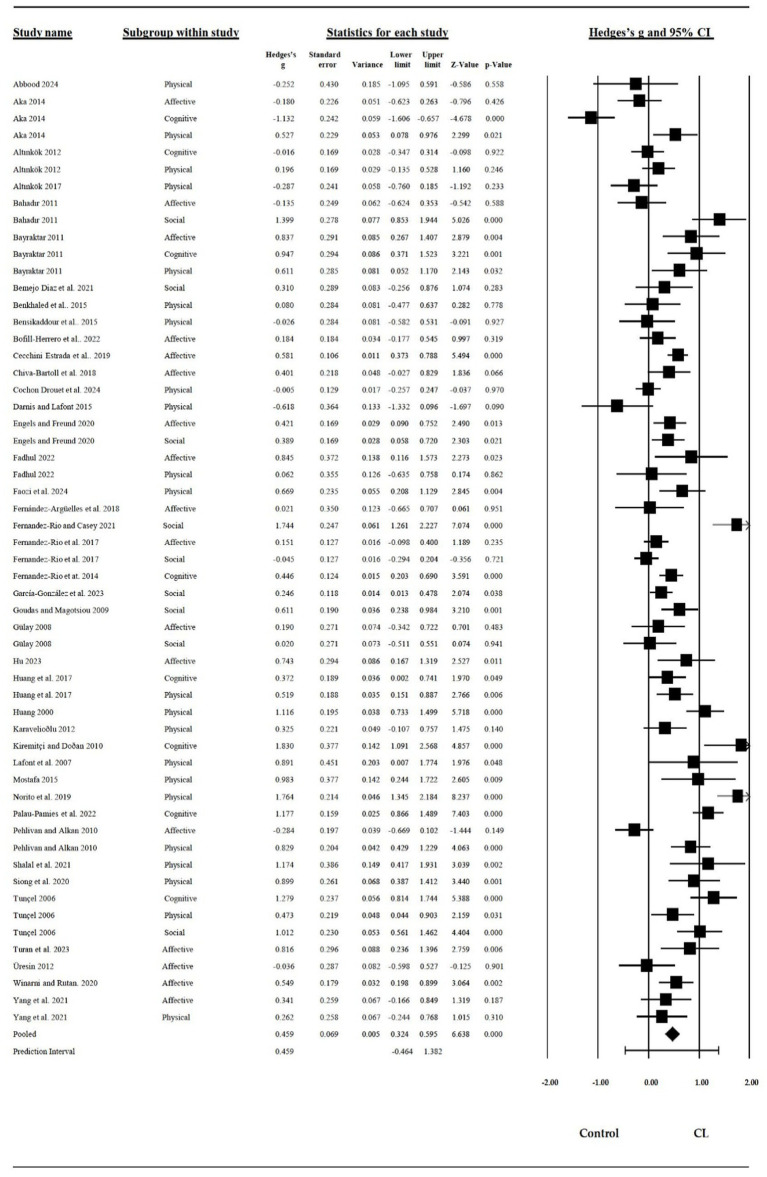
Forest plot for the random effects model.

Each report of the included studies for all continuous outcomes provided summary statistics for each group, along with an effect estimate and its precision, visualized in a structured forest plot (see Figure 3). The forest plot displays the summary statistics for each included report of the individual studies in the meta-analysis. These statistics include the ES, standard error, variance, lower limit, upper limit, z-value, and p-value for both the CL intervention and control groups. Additionally, the forest plot presents the risk ratio and its 95% confidence interval for continuous outcomes (Figure 3).

### 4.5 Results of syntheses

The present meta-analysis of 56 independent ESs, including a total of 3.985 students, yielded an overall ES: 0.459 (95% CI = 0.324 to 0.595; z = 6.64, *p* < 0.001), indicating a significant moderate positive effect of CL on learning outcomes. Cochrane’s Q was 318 and significant (*p* < 0.001, I^2^ = 82.7), suggesting heterogeneous ESs across studies (Table 4).

The subgroup analysis of 18 independent ES, involving a total of 1.919 participants, yielded an overall ES: 0.304 (95% CI [0.71, 1.107], *p* < 0.05), indicating a significant small positive effect of CL on affective learning. The subgroup analysis of 8 independent ES, involving a total of 962 participants, yielded an overall ES: 0.589 (95% CI [0.71, 1.107], *p* < 0.05), indicating a significant moderate positive effect of CL on cognitive learning. The subgroup analysis of 24 independent ES, involving a total of 1.859 participants, yielded an overall ES: of 0.471 (95% CI [0.241, 0.700], *p* < 0.001), indicating a significant moderate positive effect of CL on physical learning. Lastly, the subgroup analysis of 10 independent ES, involving a total of 1.127 participants, yielded an overall ES: 0.612 (95% CI [0.249, 0.974], *p* < 0.01), indicating a significant moderate positive effect of CL on social learning. A random-effects meta-analysis model was adopted instead of a fixed-effect meta-analysis model due to the I^2^ statistic exceeding 50% (Page et al., 2018).

#### 4.5.1 Results of additional moderator analyses

The substantial heterogeneity in ESs (as illustrated in Table 5) prompted explorations via moderator and meta-regression analyses to uncover variables potentially influencing the relationship between independent and dependent variables (Table 5). The findings of the moderator and meta-regression analyses are illustrated in Tables 3, 6.

**Table 5:** Random-model of the effect of CL and learning outcomes.

Group	k	Mean g ES	95% CI for ES	Q(g)	*I^2^*		Tau^2^	SE	Tau	z	
CL	56	0.459	[0.324, 0.595]	318.537	***	82.734	0.207	0.069	0.455	6.638	***
Affective	17	0.304	[0.140, 0.468]	40.706	**	60.694	0.065	0.084	0.256	3.633	***
Cognitive	8	0.589	[0.071, 1.107]	99.169	***	92.941	0.506	0.264	0.711	2.227	*
Physical	22	0.471	[0.241, 0.700]	105.544	***	80.103	0.227	0.117	0.477	4.023	***
Social	9	0.612	[0.249, 0.974]	66.797	***	88.023	0.261	0.185	0.511	3.306	**

CL, Cooperative learning; Index; ***p < 0.001; **p < 0.01; *p < 0.05.

**Table 6:** CL and learning outcome: univariate analyses of variance for moderator variables (categorical).

	Between-group effect (Q_BET_)	k	N	Mean g ES	SE	95% CI for ES	Homogeneity test within each group (QW)		I^2^
Source of publication	1.817								
Article		42		0.520	0.075	[0.373, 0.668]	217.533	***	81.152
Thesis		14		0.278	0.163	[−0.042, 0.598]	92.404	***	85.931
Country	0.761								
Chine		6		0.568	0.139	[0.295, 0.841]	11.541	*	56.675
Spain		11		0.470	0.134	[0.208, 0.732]	77.020	***	87.016
Türkiye		22		0.400	0.133	[0.139, 0.661]	143.066	***	85.321
Grade level	1.008								
High school		7		0.392	0.213	[−0.026, 0.810]	47.021	***	87.240
Primary school		9		0.322	0.220	[−0.109, 0.753]	66.352	***	87.943
Secondary school		24		0.534	0.098	[0.342, 0.727]	122.089	***	81.161
University		14		0.458	0.159	[0.146, 0.770]	74.512	***	82.553
Research design	1.074								
True experimental		16		0.583	0.113	[0.361, 0.804]	36.161	**	58.519
Quasi-experimental		31		0.430	0.095	[0.244, 0.616]	226.715	***	86.768
Sport content	4.877								
Basketball		12		0.637	0.112	[0.417, 0.857]	23.662	**	53.512
Combine		7		0.387	0.218	[−0.041, 0.815]	54.110	***	88.911
Physical condition		14		0.310	0.127	[0.062, 0.558]	86.902	***	85.041
Voleyball		5		0.090	0.398	[−0.689, 0.869]	53.240	***	92.487
Teaching structure	1.257								
Combine		10		0.607	0.143	[0.326, 0.888]	48.052	***	81.270
LT		6		0.473	0.231	[0.021, 0.926]	25.011	***	80.008
STAD		6		0.190	0.357	[−0.510, 0.890]	73.285	***	93.177

LT, learning team; STAD, student teams-achievement divisions; SE, standard error; CI, confidecne interval; ES, effect size; Index; ***p < 0.001; **p < 0.01; *p < 0.05.

##### 4.5.1.1 Source of publication

No significant differences in ESs were observed between articles and theses (Q_BET_ = 1.817, df = 1, *p* > 0.05; see Table 6). Therefore, the source of publication did not moderate the effect of CL.

##### 4.5.1.2 Country

No significant differences in ESs were observed among countries (Q_BET_ = 0.761, df = 2, *p* > 0.05; see Table 6). Therefore, country did not moderate the effect of CL.

##### 4.5.1.3 Grade level

No significant differences in ESs were observed among grade levels. (Q_BET_ = 1.008, df = 3, *p* > 0.05; see Table 6). Therefore, grade level did not moderate the effect of CL.

##### 4.5.1.4 Research design

No significant differences in ESs were observed between true experimental design and quasi-experimental design (Q_BET_ = 1.074, df = 1, *p* > 0.05; see Table 6). Therefore, the research design did not moderate the effect of CL.

##### 4.5.1.5 Sport content

No significant differences in ESs were observed among sports contents (Q_BET_ = 4.877, df = 3, *p* > 0.05; see Table 6). Therefore, sports content did not moderate the effect of CL.

##### 4.5.1.6 Teaching structure

No significant differences in ESs were observed among teaching structures (Q_BET_ = 1.257, df = 2, *p* > 0.05; see Table 6). Therefore, the teaching structure did not moderate the effect of CL.

##### 4.5.1.7 Duration of intervention

Meta-regression of g onto duration of intervention (in weeks) did not show significant effects of CL (Q_Model_ [1, k = 52] = 0.36, *p* > 0.05; see Table 3).

##### 4.5.1.8 Year of publication

Meta-regression of g onto publication year did not show significant effects of CL (Q_Model_ [1, k = 54] = 0.36, *p* > 0.05; see Table 3).

##### 4.5.1.9 Sample size

Meta-regression of g onto sample size did not show significant effects of CL (Q_Model_ [1, k = 52] = 0.47, *p* > 0.05; see Table 3).

##### 4.5.1.10 Study quality score

Meta-regression of g onto study quality score did not show significant effects of CL (Q_Model_ [1, k = 52] = 0.64, *p* > 0.05; see Table 3).

## 5 Discussion

The purpose of this meta-analysis was to show a quantitative synthesis on the effect of CL on students’ learning outcomes in the affective, cognitive, physical and social domains within PE context. The first research question was: (RQ1) What is the effect of CL on students’ learning outcomes in the affective, cognitive, physical, and social domains in PE? This meta-analysis, including 40 studies with 3.985 PE students and 56 ESs, revealed a significantly positive overall effect of CL at 0.459 on students’ learning outcomes in the affective, cognitive, physical, and social domains. This avant-garde quantitative summary supports the use of CL as a dynamic student-centered model that can contribute to “educationally beneficial” (p. 978) learning outcomes of PE (Kirk, 2013) with a comprehensive point of view. Moving away from meta-analytic evidences of Fernández-Espínola et al. (2020) and Liu and Lipowski (2021), which partially focuses on only one affective parameter (intrinsic motivation) but is limited by univariate methodological narrowing, limited sample population and heterogeneous dataset, this study overcomes the methodological fragmentation, which is frequently criticized in the PE literature. By quantitatively synthesizing evidence of multi-domain effects embeddedness, which has been ignored by all previous studies, the present meta-analysis adds theoretical depth to the potential of CL as a student-centered pedagogical model.

The conceptual ambiguities and implementation challenges pointed out by studies such as Fernandez-Rio and Casey (2021) and Zhou et al. (2023) are partially offset by the consistency of CL’s ESs in this meta-analysis regardless of cultural/contextual factors. For example, while Zach et al. (2023) emphasized the difficulty of implementing CL strategies by teachers, the findings of this study indicate that CL’s capacity to support student-level learning achievements is consistent despite instructional limitations, pointing to the pedagogical flexibility of the model. Furthermore, although most of the reviewed studies were based on short-term interventions (e.g., 6–8 weeks), the moderate ESs in this meta-analysis provide indirect evidence for the existence of CL’s sustained effect and advocate for a necessity of long-term research as accentuated by scholars (Casey and Goodyear, 2015; Bores-García et al., 2021).

The second research question was: (RQ2) When examining the hierarchical effects of CL on students’ learning outcomes in a cross-domain analytical framework (affective, cognitive, physical, social), how does the measurable effect trend? The meta-analytic findings revealed that the effect of CL on students’ learning outcomes exhibited statistically significant differences across learning domains in PE: The highest standardized ES was observed for social domain (0.612), followed by a decreasing trend for cognitive (ES = 0.589), physical (ES = 0.471) and affective (ES = 0.304) domains. This hierarchical distribution reinforces the notion that the collective knowledge construction mechanisms underlying CL’s superiority in social learning and the limitations in affective learning may be due to individual affective architecture deficits. With a particular emphasis on the two learning domains at opposite ends of the ES spectrum, future research should be organized along three axes: (a) dynamic neurocognitive mapping of affective-social interactions in CL processes with EEG/FNIRS-based neuroimaging, (b) scaling the effect weights of moderator variables such as affective literacy and empathic sensitivity with meta-analytic regression models, and (c) translating the affective efficacy of CL into multilayer optimization algorithms through integrated analysis of neural data streams and pedagogical big data.

### 5.1 Social learning

CL is a multidimensional pedagogical model (Casey and Kirk, 2024) that stands out for its potential to optimize and prioritize social learning outcomes in the PE context (Casey and Goodyear, 2015; Bores-García et al., 2021), but requires in-depth analysis with its methodological and epistemological layers. Meta-analytic evidence (*N* = 1.127, 10 studies) revealed that CL achieved a moderate ES (0.612) in the social domain, which was significantly superior to affective, cognitive and physical domains. This finding not only reinforces the multi-causal approach of CL in the context of PE, which places the mechanisms of social learning at the epistemic center of the pedagogical model (Casey and Goodyear, 2015; Bores-García et al., 2021; Schulze and Von Huth, 2022; Zach et al., 2023; Zhou et al., 2023); It also empirically validates the functional validity of social constructivist paradigms through (a) the optimization of role allocation in zones of proximal development, (b) the institutionalization of learning environments based on symbolic interactionism, and (c) the cognitive-affective transfer of social capital accumulation (Vygotsky, 1978). The high ES in the social domain can be explained by CL’s ontological capacity to systematically mobilize dynamics such as common goal orientation, role differentiation and social capital accumulation (Goodyear et al., 2014; Casey and Goodyear, 2015; Bores-García et al., 2021; Zach et al., 2023). However, these findings suggest that researchers and practitioners should conceptualize CL as a foundational pedagogical model strategically hybridizable with complementary frameworks, centering social learning outcomes as its core efficacy driver, rather than treating it as a universal solution detached from systemic integration. In line with the ES of 0.612 in the social domain, the team identity emphasis of the SE model (Siedentop, 2001; Siedentop et al., 2019) and the ethical leadership focus of the TPSR model (Hellison, 2011) can deepen CL’s social interaction mechanisms with social participation and social justice dimensions. Moreover, unlike the one-way communication paradigm of traditional approaches such as Mosston’s (1966) Command Style (Style A), CL’s multifocal structure stands out with its potential to automatize students’ communication skills. In this context, the sustainability of CL’s social superiority is directly related to practitioners’ ability to manage group heterogeneity.

According to Zach et al.’s (2023) discriminant analyses, in the social domain, combined strategies showed a moderate positive effect (0.402), while Jigsaw showed an unexpected negative effect (−0.403); although the classification accuracy was 79.5%, the risk of social exhaustion of long-term interventions (−0.367) emphasizes the need for contextual optimization. Furthermore, the highest ES appeared in the social domain reinforced the claim that CL has the potential to promote social capital accumulation in school settings through the synergistic dynamic of peer interaction and collective decision-making processes fostering democratic participation, multidimensional social gains such as multicultural tolerance on the axis of interpersonal and affective development, ethical empathy, and the institutionalization of compassion-oriented behavioral patterns (León et al., 2021; Ortuondo et al., 2022, 2023; Cochon Drouet et al., 2024; Kim and Park, 2024). García-González et al. (2023) demonstrated that high-structured CL significantly increased these prosocial behaviors in younger adolescents, while older adolescents showed minimal changes, underscoring age-specific and structural nuances in CL’s effectiveness. This process is not only limited to in-school interactions, but also facilitates the transfer of communicative flexibility and strategic collaboration skills (Jacobs et al., 2017; Barlett, 2019), which are among the indispensable competencies of the 21st century, to out of school settings based on the principle of interdisciplinary permeability, thus acting as a catalyst for sustainable social transformation. From this point of view, it may be asserted that CL can be used as a pedagogical antidote to problems such as peer bullying. However, a theoretical counter-thesis claiming that the educational value of CL should be sought in motivational dynamics rather than social learning outcomes (Montoya et al., 2020) points to the epistemological paradox of this approach, warning that the instrumentalization of self-determined motivation (Sierra-Díaz et al., 2019) and intrinsic motivation (Fernández-Espínola et al., 2020; Liu and Lipowski, 2021) may undermine pedagogical integrity. This dialectical tension creates a critical debate on how to strike a balance between the social effects of CL and its motivational underpinnings in CL-based program designs. In this context, CL implementation should balance social gains with students’ intrinsic motivation and avoid overly structured group work.

The transformative role of CL in the context of PE goes beyond mere instrumental skills, emerging as a multi-layered pedagogical model intertwined with a sociomoral ethical framework. Embedded within this model-based approach (Casey and Kirk, 2024), as Dyson et al. (2004) emphasize, is the synergy between the dynamics of interpersonal interaction and high-quality social gains. This synergy supports the development of functional skills such as the exchange of ideas, the construction of shared meaning and active listening (André et al., 2011; Bensikaddour et al., 2015), while at the same time building a sociomoral paradigm based on fairness, mutual responsibility and collective belonging, as Wallhead and Dyson (2017) and Sánchez-Hernández et al. (2018) point out. Casey and Goodyear’s (2015) conceptualization of ‘pedagogical empathy’ embodies this dual structure, suggesting that CL functions as a bridge that nurtures mechanisms of mutual care and respect support among students.

In essence, this multidimensional architecture of CL combines the instrumental (skills-based) and ethical (value-oriented) paradigm into a synergistic whole, as suggested by Dyson et al. (2020). This synthesis not only supports affective, cognitive, and physical development in a holistic axis, but also transforms PE into a ‘social microcosm’ by reconstructing learning processes with democratic participation, social justice and ethical sensitivity. Thus, beyond being a methodological tool, CL functions as an epistemological framework that transforms both the individual and collective identity construction of students.

### 5.2 Cognitive learning

A subgroup analysis of eight studies involving 962 students revealed that CL had a moderate positive effect on cognitive learning outcomes. This impact was evidenced by a modarate ES of 0.589, ranking as the second-highest among the analyzed results. This synthesized quantitative evidence aligns with studies demonstrating that CL facilitates cognitive learning, including the ability to analyze team strategies, enhance decision-making and problem solving skills, and engage in meaning-making activities (Dyson et al., 2016; Gorucu, 2016; Bodsworth and Goodyear, 2017; Fernández and González-Mesa, 2018; Nopembri et al., 2019).

Although the ES of CL in the cognitive domain (0.589) makes sense with peer-mediated knowledge transfer and modeling processes in Bandura’s (1986) social cognitive theory, its low value compared to the social domain (ES = 0.612) may be due to CL’s ontological focus on shared meaning construction while marginalizing individual cognitive deepening. However, this quantitative finding proves its practical relevance in real classroom settings through critical dynamics. CL’s structured group interactions increase the pedagogical intensity of lesson time by enabling multiple simultaneous learning outcomes (social-cognitive; O’Leary and Griggs, 2010; Casey and Goodyear, 2015) compared to teacher-centered approaches. For example, in a basketball tactical analysis activity, CL groups parallel strategic decision-making (cognitive) and peer feedback (social) processes, enabling double gains in a single class period. The heterogeneous group structure (Slavin, 1990) turns students’ cognitive style differences (analytical-intuitive) into an advantage; when hybridized with TGfU, CL’s social dynamics strengthen inclusiveness by enabling tactical problem solving skills (Bunker and Thorpe, 1982; Ortiz et al., 2023), strategic engagement of even low motor-skill students. The significance of ES = 0.589 lies in the fact that CL promotes not only cognitive gains but also 21st century skills such as social–emotional learning and ethical reasoning (Dyson and Casey, 2016). CL practices integrated with Mosston’s (1966) Exploratory Style (Style E) expose students to multi-layered cognitive-social processes such as open-ended hypothesis testing and managing in-group conflicts of opinion. This triple synergy transforms CL’s ES = 0.589 from a mere statistical output to an indicator of instructional sustainability. The critical point is that researchers and practitioners should adopt structured flexibility, adapting the social framework of the CL in such a way as to personalize the levels of cognitive challenge according to student profiles. For example, the cyclical distribution of roles such as observer-strategist and practitioner-critic within CL units in a PE lesson nurtures both motor skill development and meta-cognitive awareness. In this regard, the cognitive ES value of CL is evidence of its capacity to function as a cognitive catalyst of social learning, but this potential only becomes possible when instructional design is realized by practitioners who can manage the dialectic between quantitative data and classroom ontology.

Vygotsky’s (1978) conceptualization of ‘the zone of proximal development’ radically illuminates the cognitive developmental mechanisms of CL: Peer feedback and collective knowledge construction, not only through ‘more knowledgeable others’ that exceed individual capacity, but also by creating distributed cognitive systems (Hutchins, 1995), enable learners to socially negotiate meaning through symbolic means (language, argument structures). In this regard, it can be emphasized that the ‘the zone of proximal development’ is not a static territor but a dynamic space of interaction (De Costa, 2007). For example, conflicting perspectives in CL groups may force students to experience cognitive contradiction (Piaget, 2013), triggering the restructuring of existing schemas. Critical here, however, is how Vygotsky’s emphasis on social interaction synergizes with Flavell’s (1979) model of metacognitive regulation: CL should provide students with not only strategic decision-making (e.g., resource allocation or task division) but also socio-metacognitive awareness (Salomon and Perkins, 1998)—that is, the ability to ‘think about how we think as a group’.

The critical role of cognitive learning in CL was examined by Gorucu (2016) in the context of constructivist pedagogy, emphasizing that problem-solving activities develop students’ ability to build collective intelligence and cognitive adaptation through interdependence dynamics. In the samle line, Bodsworth and Goodyear (2017) study, which draws attention to the potential of digital tools within PE context to instrumentalize reflective learning environments in CL. The researchers argued that technology deepens students’ capacity to ‘learn how to learn’ by triggering not only knowledge transfer but also meta-reflection (high-level analysis of the learning process) and epistemic curiosity (motivation to access knowledge). This dynamic nature of CL is explained in Bores-García et al.’s (2021) systeamtic review through the concepts of active subjectivity and structured reflection. For example, students are forced to question their own thinking strategies and gain cognitive flexibility while solving complex problems through social interaction. This can be explained by the highly reflective components inherent in CL tasks (e.g., argument revision in group discussions or iterative testing of the solution prototype). The student’s participation in the process of CL as an ‘actor’ fosters not only knowledge acquisition but also self-regulated learning (Zimmerman, 2002) and socio-cognitive conflict resolution skills.

In the context of PE, to systematically operationalize the effect of CL on cognitive learning outcomes in a measurable and feasible framework, researchers and practitioners should integrate systemic thinking (Senge, 2006) and epistemic cognition (Chinn et al., 2014) when designing interdisciplinary problem scenarios; and develop scientific logic and social negotiation skills through reasoning diagrams in unconstrained problems such as climate change. In digital hybridization strategies, using the principles of cognitive load (Sweller, 1988), social network analysis (Wasserman and Faust, 1994), and visual collaborative brainstorming with tools such as Padlet or Miro can optimize distributed attention management by visualizing the flow of ideas within the group. Metacognitive reflection mechanisms should adopt the perspective of double loop learning (Argyris and Schön, 1997), focusing on the questions ‘what did we do?’ (first loop) and ‘how did we think?’ (second loop) in group communications and bridging Zimmerman’s (2002) self-regulation cycle (planning-monitoring-evaluation) between individual and collective reflection. However, social epistemology (Goldman, 2019) emphasizes the construction of knowledge in a network of social practices, transforming education into a process in which ‘communities of learners’ (Lave and Wenger, 1991) pursue common goals; this transformation requires understanding CL not as a technical tool but as a pedagogy focused on democratic participation and critical consciousness (Freire, 1970).

### 5.3 Physical learning

In the subgroup analysis covering 24 studies with a total of 1.859 students, it was found that CL had a low-to-moderate positive effect on learning outcomes in the physical domain, demonstrading with the third-largest ES (0.471). However, this finding is valuable and functional even under realistic school conditions. Under time constraints, CL optimizes multiple gains by integrating physical skill development with social interactions (role distribution, peer feedback; Dyson and Casey, 2016). In heterogeneous classrooms (Slavin, 1996), inclusive participation is promoted with high and low ability students taking on model-leader and observer-learner roles. For non-expert teachers, CL compensates for technical deficiencies with social self-management by providing a pedagogical template with clear role allocations (Casey and Goodyear, 2015). This effect is in line with the goal of sustainable efficiency in less than ideal conditions. Therefore, CL’s moderate effect triggers indirect physical gains such as social capital accumulation and motivational boosts even in resource constraints. In addition, this finding confirms the role of CL in enhancing motor skill development and physical performance, while demonstrating a significant superiority compared to traditional individual and competitive pedagogical approaches. Casey and Goodyear (2015) asserted that within CL-oriented group discussions, students’ deliberative engagement in the co-construction of movement techniques not only stimulates metacognitive consciousness but also transmutes the interactive process into a dynamic, self-reinforcing synergy. This emergent pedagogical phenomenon, they argue, operates through a dialectical interplay wherein the iterative refinement of physical praxis (e.g., kinesthetic precision, proprioceptive calibration) reciprocally scaffolds the evolution of strategic cognitive schemata (e.g., problem-solving heuristics, adaptive decision-making frameworks). In this context, the comparatively attenuated ES of CL in the physical domain (0.471) may be epistemologically rooted in the neurobiological and biomechanical exigencies inherent to motor skill acquisition—specifically, the non-negotiable role of individualized repetition, proprioceptive feedback loops, and task-specific neuromuscular plasticity. This ontological constraint positions CL not as a pedagogical panacea but as a contingent adjunct, necessitating strategic integration within frameworks prioritizing automatization. For instance, the SE model’s symbiotic incorporation of CL (e.g., Fernandez-Rio and Casey, 2021) could theoretically engender a dual-axis developmental matrix, harmonizing technical skill refinement with collective tactical coherence through socially embedded praxis. Analogously, Mosston’s (1966) Practice Style (Stil B), with its regimented iterative scaffolding, might serve as a counterbalance to CL’s dialogic dynamism, creating a dialectical pedagogy oscillating between mechanistic precision and social constructivism. However, such hybridization demands temporal granularity and hierarchical task architecture from educators; failure to calibrate these variables risks metastasizing into pedagogical dissonance, wherein socio-cognitive and sensorimotor objectives engage in zero-sum competition. Crucially, in contexts marred by temporal constraints, heterogeneous skill stratification, and reliance on generalist instructors, this duality becomes exponentially precarious. Without scaffolded curricular blueprints or micro-sequenced progressions, non-specialist educators may inadvertently dilute both social cohesion and skill mastery, rendering CL’s integration a performative compromise rather than a synergistic augmentation.

With regard to physical learning, CL is conceptually grounded in the synergistic interplay of peer interaction that fosters knowledge co-construction (e.g., Dyson et al., 2016), schema optimization that enhances cognitive adaptation (Schmidt, 1975), social motivation that promotes goal-directed engagement (Bandura, 1986; Bandura, 1986), and the mirror-neuron system that activates motor responses through observation (Rizzolatti and Craighero, 2004). In accordance with these theoretical accounts, a meta-analytic review by Stanne et al. (1999) found that peer-assisted learning (not embedded within a direct CL framework) demonstrated a statistically significant superiority in pedagogical effectiveness on movement skill acquisition (ES = 0.53) compared to individual effort-based or competitive instructional models (ES = 0.36). Furthermore, studies examining the effects of CL on physical learning present a panorama shaped by methodological pluralism but with some epistemological gaps. Quantitative approaches (Lafont et al., 2007; Luo et al., 2013; Benkhaled et al., 2015; Darnis and Lafont, 2015; Altınkök, 2017; Huang et al., 2017) have statistically validated the role of CL in social skill development - through dynamics such as dialog, listening and collective understanding building - emphasizing the positive correlation of physical activity with motor skills and social bonds, but compressing the unmeasurable phenomena of body expression (emotional codes of gestures, bodily metaphors) into a reductionist template. In contrast, qualitative studies (Barrett, 2005; Callado et al., 2014; Bensikaddour et al., 2015) have revealed the contextual depth of CL in physical interactions and explained how individuals’ bodily experiences are intertwined with belonging, self-confidence and creative expression through micro-ethnographic analyses, but have been limited to small samples whose generalizability has been questioned. Although mixed-method research (Lee, 2014) blends the structural rigor of quantitative data with the narrative richness of qualitative findings in an attempt to overcome this dichotomy, revealing the multidimensional effects of CL (social learning reflected in motor skills, group synchronization fostering emotional resilience), it fails to place the symbolic language of the body (creative physical communication, performative expression) in a systematic theoretical framework, thus the hegemonic focus on sport and motor skills overshadows the potential of the body as a social-affective signifying tool. This methodological fragmentation coincides with Bores-García et al. (2021) critique, pointing out that CL practices, by constructing the body only as a technical object, neglect its constitutive and transformative role in social interaction, and that this deficit narrows critical dimensions such as creativity, identity construction and cultural diversity in educational processes. This deficiency can be reconsidered with the critical argument from Merleau-Ponty’s (2012) bodily phenomenology perspective that motor skills are not a mere technical acquisition but represent a form of bodily consciousness integrated with social-cultural practices.

### 5.4 Affective learning

The subgroup analysis of the present meta-analysis covering 18 studies with a total of 1.919 students revealed that CL had a low positive effect on learning outcomes in the affective domain (ES = 0.304). This finding is somewhat consistent with the low ES of intrinsic motivation (0.380) found by Fernández-Espínola et al. (2020) and Liu and Lipowski (2021), indicating that the CL has limited but statistically significant potential to enhance students’ affective learning within different PE settings. We believe that the seemingly low ES is not a superficial weakness, but may be due to the long-term, time-consuming nature of affective development that requires contextual support. This may also stem from a misalignment between collective group dynamics (collective synergy) and individual affective processes (affective architecture). In particular, the autonomous nature of intrinsic motivation, the person-specific trajectory of self-efficacy, and the cognitive isolation of affective self-regulation may play a critical role in determining the ontological boundaries of CL. This paradox may force CL into a radical hybridization with the TPSR model. TPSR’s ethical autonomy and self-esteem-oriented framework can combine CL’s social interaction with layers of affective signification in a fragile synchrony (Hellison, 2011; Shen et al., 2022). Dewey (1986)‘s principles of experiential learning and Rogers’ person-centered approach can provide affective deepening by integrating emotional diaries or self-reflection protocols into CL processes. Mosston’s (1966) Individual Program Style (Style I) brings the group dynamics of CL into dialectical tension with affective individuality, reconciling collective belonging and personal interiority on a fragile equilibrium. However, in contexts where empathic leadership skills are not systematically instilled, this integration may risk turning affective superficiality and social performativity into a symbiotic vicious circle. Especially when working with groups of students with low affective literacy and educators lacking pedagogical sensitivity, the affective integration of CL can be reduced to an artificial ‘theater of emotions’, turning the effect into an ontological illusion.

Wang (2012) explained the transformative effect of CL on dynamics such as motivation and self-confidence by synthesizing Johnson and Johnson’s (2009) and Slavin’s (1996) views, emphasizing that especially collective achievement experiences and synergistic goal settings within the group increase students’ intrinsic motivation. When contextualized within Bandura’s (1986) social cognitive theory it is seen that peer feedback triggers self-efficacy construction and individual motivation is integrated into collective goals through social modeling. However, the way CL triggers affective mechanisms is indirect rather than direct; Zach et al. (2023), while proving the link of CL’s Jigsaw and Combined strategies with affective outcomes, pointed out that Learning Teams produced this association relatively fewer, drawing attention to the need for pedagogical design to be goal-oriented. This is consistent with Deci and Ryan’s (2000) self-determination theory: CL’s low ES may be due to a failure to balance the needs for autonomy, competence and relatedness. For example, the motivation of ‘unenthusiastic’ students in leadership roles observed by Goodyear et al. (2014) can be explained by supporting autonomy, while Slavin’s (1996) goal structuring meets the need for competence and Johnson and Johnson’s (2009) positive dependency principle meets the need for relatedness.

The limited effect potential of CL is further complicated by methodological challenges: Casey and Goodyear’s (2015) critique of ‘anecdotal evidence’ points to the shallowness of studies that neglect the temporal variability of affective learning and the role of cultural context. Although the large sample (*N* = 1.919) and heterogeneous subgroups of the meta-analysis partially explain the inconsistencies, social desirability bias in self-report measures and cross-sectional designs stand out as factors that suppress ES. These methodological gaps are also related to Vygotsky’s (1978) concept of zone of proximal development: Group interactions allow students to internalize their affective development through ‘peer scaffolding’, but cross-sectional studies cannot capture the temporal dynamics of this process. Dyson et al. (2020) asserted that the developmental dynamics of the sociomoral effects of CL (fairness, active listening) cannot be fully grasped due to the lack of longitudinal evidence.

The role of cultural context on the effectiveness of CL is critical in Hofstede’s (2011) collectivism–individualism tension axis. Bronfenbrenner’s (1979) ecological systems theory further deepens this context: In collectivist cultures, affective learning can be enhanced when the internalization of group goals (macrosystem) is aligned with the learner’s role models in society (mesosystem). Walad et al. (2019) observed that the Jigsaw strategy breaks the dominance of powerful students and ensures equal participation, leading to progress in social communication and attitude development. This finding is in line with Freire’s (1970) critical pedagogy perspective: In-group role distribution can transform power dynamics by offering students who are marginalized in traditional settings (e.g., female students) an ‘emancipatory space for dialog’. Casey and Fernandez-Rio (2019) contended that how this effect differs in the context of cultural heterogeneity should be tested with models of glocal adaptation.

In addition to the CL’s low ES in the affective domain (0.380), the subjective, personal, multidimensional, and complicated nature of affective learning (Pope, 2005) reveals the need for hybrid pedagogies beyond traditional CL models. The integration of social–emotional learning (SEL) and CL can create an ecosystem that nurtures self-evaluation and social justice perspectives; however, the success of this integration depends on restructuring teacher education to support the affective domain of CL. The learning outcomes in the affective domain of CL link with Seligman’s (2011) five domains of well-being: Positive Emotions, Engagement, Relationships, Meaning, and Accomplishment (PERMA): Group synergy (Relationships) and collective achievement (Achievement) can trigger meaningful learning (Meaning) and pleasure or elusive feelings (Positive Emotion) in students. O’Leary and Griggs (2010) and Baena-Morales et al. (2020) emphasized the need for integrated frameworks that center intrinsic motivation, self-efficacy, and perceptions of social justice rather than technical models of collaboration for this optimization. Consequently, the affective potential of CL can only be unlocked through interdisciplinary perspectives, methodological depth, and contextual sensitivity.

The third research question was: (RQ3) How are the ES of CL affected by moderator variables such as publication source, country, grade level, research design, sport content, teaching structure, intervention duration (weeks), publication year, sample size and study quality score (a unique variable)? In this study, the ES of CL did not differ significantly (*p* > 0.05) in any of the moderator variables examined, suggesting that the structural components of CL’s pedagogical effectiveness are based on the epistemological foundations of social constructivism theory rather than contextual or methodological conditions. As highlighted by Casey and Goodyear (2015) and Bores-García et al. (2021), the effect of CL on learning outcomes in the affective (anecdotally), physical, cognitive and social domains is a reflection of the model’s universal principles. This is consistent with similar study findings being observed across different geographies (e.g., conceptual uncertainties in China, Dyson et al., 2022) or developmental stages. However, the operational reductionist definition of moderators (e.g., broad categorization of ‘sport content’) may have masked the influence of context-specific dynamics (cultural collectivism, implicit teacher competence), creating an artificial impression of consistency.

The non-significant meta-regression results for intervention duration and sample size (Q > 0.05) suggest that the mechanisms of action of CL may be independent of time or scale, but related to critical thresholds (e.g., minimum time required for the formation of social cohesion). This finding is in line with Fernández-Espínola et al.’s (2020) and Liu and Lipowski’s (2021) warnings about heterogeneity in intrinsic motivation, revealing the limits of measuring CL’s non-linear pedagogical dynamics (initial rapid social adaptation, followed by plateaus) with traditional linear models. Consequently, this coherence paradigm highlights the potential of CL as a transdisciplinary pedagogy, necessitating the use of micro-moderators (e.g., teacher-student trust index, cultural collectivism score) and neurocognitive measures (neural correlates of joint attention) in future research. The highly controlled hybrid designs proposed by Zach et al. (2023) and Zhou et al. (2023) could bridge the epistemic gap between theory and practice by testing the contextual flexibility of CL through a dialectical synthesis between the Vygotsky’s (1978) ‘Zone of Proximal Development’ and the Gibson’s (1986) Affordances Theory.

### 5.5 Limitations and future research directions

The findings from this meta-analysis reveal a methodologically sound body of research on the effect of CL on students’ learning outcomes in PE. However, there are a number of critical issues that need to be identified to fill the gaps in the literature. Due to the authors’ mastery of only English, Spanish and Turkish, this study excludes publications in other languages. The inclusion of multilingual research teams in future systematic reviews and meta-analyses may contribute to enriching the CL literature with interdisciplinary and intercultural perspectives. Furthermore, there is a need for more comprehensive analyses to elucidate the causal relationships underlying the mechanisms of CL’s effect on PE learning outcomes (affective, cognitive, physical, social). In this context, structural equation modeling such as critical path analysis can be used as a methodological tool to uncover causal links between knowledge components and CL interventions. The meta-synthesis method that can be applied to integrate qualitative findings will allow for a multidimensional conceptualization of the phenomenon.

There can be a significant time gap between data collection and publication, which increases the risk of systematic bias in trend analyses based on publication year. To address this methodological weakness, it is essential that researchers transparently report the chronology of data collection and that journal editorial policies enforce this standard. Such regulations would constitute an epistemological step that would increase the validity of literature reviews examining temporal dynamics. Although the CL studies were conducted in 12 different countries (Algeria, China, Egypt, France, Germany, Greece, Indonesia, Iraq, Malaysia, Spain and Türkiye), the limited geographical and cultural diversity makes the universal generalizability of the findings questionable. Comparative studies in different sociocultural contexts are needed to reach globally valid conclusions. However, the current methodological limitations do not invalidate the main arguments of this meta-analysis.

## 6 Conclusion

The ES hierarchy of the CL model on students’ learning outcomes in the social (ES = 0.612) > cognitive (ES = 0.589) > physical (ES = 0.471) > affective (ES = 0.304) domains within different PE contexts systematically shows how CL’s social constructivism-based epistemology transforms learning processes, particularly through the mechanisms of peer dialog and collective goal orientation. In light of these findings, opportunities emerge for teachers to strengthen collaboration by developing pedagogical designs that center on social interaction; for curriculum developers to build interdisciplinary programs that optimize learning outcomes through the collective synergy of CL; for policymakers to ground school climate policies that support multiple development in a hierarchical context with CL’s empirical findings; and for researchers to deeply analyze the underlying causes of ESs (e.g., high ES in social learning or low ES in affective learning). This essay offers divers stakeholders the possibility to restructure educational ecosystems with strategic interventions based on quantitative data and to integrate the principles of social constructivism at the micro–macro level. Thus, the spectrum of CL’s effect transcends theoretical assumptions and provides the analytical ground for a scalable and sustainable transformation in PE practice.

## Data Availability

The original contributions presented in the study are included in the article/supplementary material, further inquiries can be directed to the corresponding authors.
